# Rheo-SAXS study on electrically responsive hydro­gels with shear-induced conductive micellar networks for on-demand drug release

**DOI:** 10.1107/S1600576725002808

**Published:** 2025-04-25

**Authors:** Thuy Thien Ngan Vo, Yi-Wei Chang, Chun-Jen Su, U-Ser Jeng, Chih-Chia Cheng, Ya-Sen Sun, Wei-Tsung Chuang

**Affiliations:** a Graduate Institute of Applied Science and Technology National Taiwan University of Science and Technology Taipei106335 Taiwan; b Department of Chemical and Materials Engineering National Central University Taoyuan32001 Taiwan; c National Synchrotron Radiation Research Center Hsinchu300092 Taiwan; d Department of Chemical Engineering National Tsing Hua University Hsinchu300044 Taiwan; e Department of Chemical Engineering National Cheng Kung University Tainan70101 Taiwan; Brazilian Synchrotron Light Laboratory, Brazil

**Keywords:** micellar crystals, hydro­gels, electrically stimulated drug release, rheo-SAXS

## Abstract

This study presents a novel approach to creating electrically responsive hydro­gels utilizing a poly(ethyl­ene oxide)–poly(propyl­ene oxide)–poly(ethyl­ene oxide) (PEO_100_–PPO_65_–PEO_100_) triblock copolymer, functionalized with benzene­sulfonate end groups to form sF127. This functionalization allows the incorporation of sF127 into F127 micelles, resulting in tailored micelles designated as F_18_S_2_P when combined with poly(3,4-ethyl­ene­dioxy­thio­phene):poly(benzene­sulfonate) (PEDOT:PSS). For comparison, a control system using non-functionalized PEDOT:PSS/F127 micelles, designated F_20_S_0_P, was also developed. Using piroxicam as a model hydro­phobic drug, we evaluated the hydro­gel’s drug encapsulation efficiency and electrical responsiveness. The functionalized F_18_S_2_P hydro­gel demonstrated superior performance of electrically stimulated drug release, especially when prepared with a blade-coating process. *In situ* rheological small-angle X-ray scattering (rheo-SAXS) measurements under large amplitude oscillatory shear revealed that function­alization facilitates crystal plane sliding, leading to the formation of a randomly hexagonal close-packed (rHCP) sliding layer structure. This behavior contrasts with the face-centered cubic to rHCP phase transition observed in the unfunctionalized hydro­gel. *In situ* SAXS analysis under applied electric fields (E-SAXS) further confirmed the electroresponsive micellar deformation. By integrating the rheo-SAXS and E-SAXS findings with blade-coating processing insights, we identify a clear structure–function relationship that governs the performance of these hydro­gels. The enhanced drug delivery of the function­al­ized F_18_S_2_P hydro­gel is attributed to the electrostatic attraction between the positively charged PEDOT and the negatively charged benzene­sulfonate-functionalized micelles. This interaction creates conductive nanonetworks within the hydro­gel, significantly improving its ability to release drugs in response to electrical stimulation. This work highlights the potential of electrically responsive hydro­gels for precise, localized drug delivery applications.

## Abstract 2

In this study, electrically responsive hydro­gels were developed with enhanced on-demand drug release by combining poly(3,4-ethyl­ene­dioxy­thio­phene):poly(benzene­sulfonate) with Pluronic F127 micelles functionalized with negatively charged benzene­sulfonate groups, forming conductive nanonetworks. Evaluations using piroxicam and advanced structural analyses (including rheological small-angle X-ray scattering, rheo-SAXS) revealed that blade-coating fabrication improves conductivity and voltage-triggered drug release efficiency, showcasing the potential for on-demand transdermal drug delivery patches.

## 1. Introduction

Smart drug delivery systems (SDDSs) primarily aim to enhance the pharmacological activity of drugs, increase drug loading capacity and achieve controllable release timing for precise delivery to target areas (Akhoon, 2021 ▸). This innovative therapeutic technology addresses challenges such as poor drug solubility, low bioavailability and adverse side effects. To do so, the development of stimulus–response materials for drug carriers essentially achieves precision medicine via precise control over the timing and concentration of drug release, thereby maximizing therapeutic outcomes (Benoit *et al.*, 2020 ▸; Vargason *et al.*, 2021 ▸).

Hydro­gels are increasingly recognized as essential drug carriers in SDDSs due to their unique structure and properties (Correa *et al.*, 2021 ▸; Yuk *et al.*, 2019 ▸). As 3D hydro­philic polymer networks, hydro­gels can retain large amounts of water, creating a soft, tissue-like consistency that makes them highly compatible with biological tissues (Hu *et al.*, 2019 ▸). This biocompatibility enables hydro­gels to serve as effective carriers for drugs, allowing for controlled, localized and sustained release of therapeutic agents when triggered by external stimuli such as temperature, pH, electric fields and light (Lavrador *et al.*, 2020 ▸). Additionally, hydro­gels can encapsulate a wide range of molecules, from small drugs to large biomolecules (Farasati Far *et al.*, 2024 ▸). This encapsulation can protect drugs from degradation, improve bioavailability and enhance the solubility of poorly soluble drugs. Another advantage of hydro­gels in drug delivery is their adaptability during the 3D-printing process and their ability to be safely implanted, released and ultimately degraded (Zhang & Wang, 2022 ▸; Askari *et al.*, 2021 ▸). These ‘smart’ properties enable hydro­gels to release drugs at specific times or under particular conditions, providing tailored treatment options that increase therapeutic effectiveness while minimizing side effects. With these capabilities, hydro­gels play a crucial role in advancing SDDSs, offering personalized treatment options and improving patient outcomes.

Pluronic F127 is a non-toxic FDA-approved triblock co­poly­mer composed of hydro­philic poly(ethyl­ene oxide) (PEO) and hydro­phobic poly(propyl­ene oxide) (PPO) arranged in a PEO–PPO–PEO structure (Kabanov *et al.*, 2002 ▸). This amphiphilic configuration enables F127 to form core–shell micelles at concentrations above its critical micelle concentration of approximately 16 wt% and temperatures above its critical micelle temperature of 12°C (Wanka *et al.*, 1994 ▸). The unique thermoresponsive and biocompatible micelles can also encapsulate hydro­phobic drug molecules within the PPO core to enhance the solubility of poorly water-soluble drugs and protect them from degradation, improving their stability and bioavailability (Nie *et al.*, 2011 ▸). Another advantage of F127 is its ability to undergo a reversible sol-to-gel transition near body temperature. At cooler temperatures, F127 solutions remain in a liquid state, facilitating easy injection. On warming to physiological temperatures, F127 rapidly gels, creating a stable matrix that enables controlled and sustained drug release at the target site. This temperature-responsive property is particularly valuable for localized drug delivery, as it allows for direct application to the treatment area and minimizes systemic side effects. Therefore, many researchers have functionalized the end groups of F127 chains to achieve a broader range of stimulus–response materials in SDDS applications (Singh *et al.*, 2019 ▸; Adepu & Ramakrishna, 2021 ▸; Cao *et al.*, 2021 ▸; Xie *et al.*, 2021 ▸; Jin *et al.*, 2017 ▸).

Among the stimulus–response materials, electrically responsive hydro­gels stand out due to their ability to provide on-demand drug release through wearable electronic devices (Puiggalí-Jou *et al.*, 2019 ▸; Murdan, 2003 ▸; Kolosnjaj-Tabi *et al.*, 2019 ▸; Boehler *et al.*, 2019 ▸). The conductive properties of conducting polymers arise from their conjugated π-systems, which enable electron transport through redox reactions. Therefore, adding conducting polymers to these hydro­gels can alter their physical structure, for example by making them swell or contract to control the release of drugs at specified rates and dosages, and can cause them to undergo electrically induced redox reactions (Mirvakili & Langer, 2021 ▸; Mrinalini & Prasanthkumar, 2019 ▸; Murdan, 2003 ▸). Although the F127 hydro­gel has excellent biocompatibility and capability for drug release, the primary focus of this work was to further develop the hydro­gel into an electrically responsive drug delivery hydro­gel with precise, on-demand drug release characteristics.

In this study, based on findings related to the potential of conductive hydro­gels in drug delivery, we developed electrically responsive hydro­gels as drug carriers using hybridized poly(3,4-ethyl­ene­dioxy­thio­phene):poly(benzene­sulfonate) (PEDOT:PSS) and functionalized F127 micelles. To achieve this, negatively charged surfaces were introduced to the F127 micelles through partially benzene­sulfonate-terminated F127 (designated as sF127). This modification allowed positively charged PEDOT to envelop the micelles, forming a conductive nanonetwork within crystalline-packed micellar hydro­gels. As a model hydro­phobic drug, we used piroxicam (PX) to assess the drug loading and drug release capabilities of the conductive hydro­gel triggered by electricity. Through *in situ* rheological small-angle X-ray scattering (rheo-SAXS) measurements and SAXS analysis under applied electric fields (E-SAXS), we reveal a shear-driven randomly hexagonal close-packed (rHCP) structure that facilitates the formation of uniform, conductive nanonetworks around sulfonated micelles in the hydro­gel. Shear-induced micellar reorganization facilitates more effective voltage-driven redox interactions, thereby promoting micelle deformation and enhancing drug release. Importantly, this shear-induced structural transformation is replicated through the blade-coating process, linking fabrication stress conditions to functional performance.

## 2. Experimental

### 2.1. Sample preparation

Pluronic F-127 with a molecular weight of 12600 and PEDOT:PSS were purchased from Sigma–Aldrich (USA). PX was purchased from Combi-Blocks Co. Drug encapsulation was achieved using the thin-film hydration method. Conductive hydro­gel drug release samples were prepared using carbon felt substrates measuring 1.5 cm in diameter and 3 mm in thickness and by either immersing them in the hydro­gel solution or applying a blade-coating of hydro­gel to ensure infiltration into the carbon felt structure.

### 2.2. Instrumentation

The rheo-SAXS measurements were conducted at beamline 23A of the Taiwan Light Source (TLS) at the National Syn­chrotron Radiation Research Center (NSRRC), Taiwan (Jeng *et al.*, 2010 ▸). Rheological testing was performed using an Anton Paar MCR 501 rheometer equipped with a polycarbonate Couette cylinder cell featuring a 1 mm gap and a temperature-controlled stage. The incident X-ray beam, with a wavelength of λ = 0.8265 Å, was directed along the radial and tangential directions of the Couette cylinder cell for obtaining 2D SAXS patterns. The rheo-SAXS experimental setup is depicted in Fig. S1(*a*). The Couette cylinder cell holds approximately 5 ml of sample. Samples were introduced into the cell at 4°C to maintain a liquid state and then allowed to stabilize at 25°C for 1 h to form a gel. Rheological measurements were performed in large amplitude oscillatory shear (LAOS) mode at a fixed frequency of 1 Hz, with strain sweeping from 0.1 to 500%. SAXS data were simultaneously recorded at each strain point. The 1D SAXS profiles, *I*(*q*), were integrated across the entire azimuthal angle in the measured *q* range, where the scattering vector magnitude, *q* = 4π sin θ λ^−1^, was determined by the X-ray wavelength λ and the scattering angle 2θ.

Fourier-transform infrared spectroscopy (FTIR) was conducted at beamline 14A of the TLS at NSRRC, Taiwan. Differential scanning calorimetry (DSC) measurements were carried out using a Diamond DSC system (PerkinElmer) with a programmed heating and cooling rate of 10°C min^−1^. NMR spectra were obtained using a Bruker AVIII HD-600 NMR spectrometer. UV–vis spectroscopy was performed on JASCO instruments (V-670 and ARSN-733). The gel’s conductivity was assessed using a potentiostat (CHI627E, CH Instruments) and impedance spectroscopy (SI 1260, Solartron Analytical). The conductivity of the hydro­gel was measured using electrochemical impedance spectroscopy (Biologic SP-150e) in a Swagelok cell over the frequency range 100 mHz to 500 kHz.

## 3. Results

### 3.1. Synthesis of sunfonic-F127 micelles

**Figure 1:**
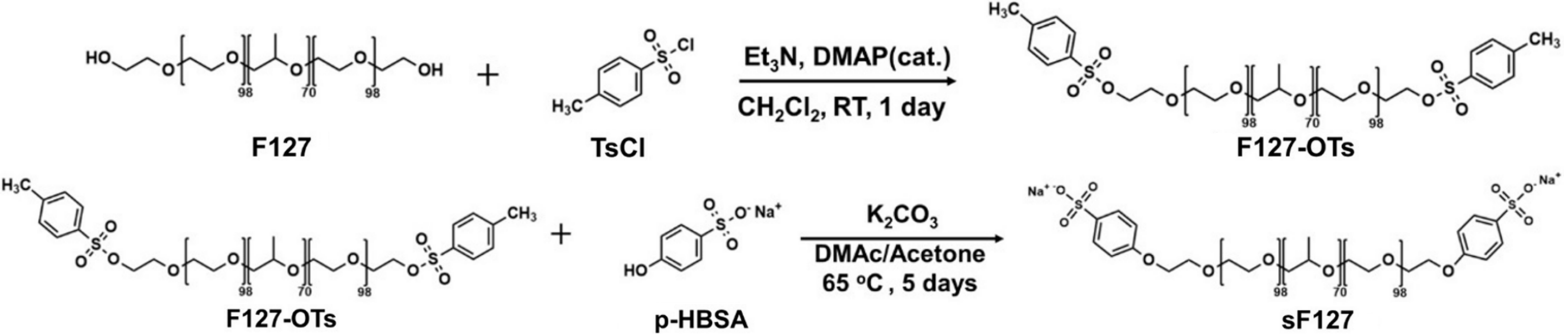
Chemical synthesis of sF127.

The conductivity of the hydro­gel is strongly associated with drug release efficiency. The blending process, wherein a conductive polymer is blended with a non-conductive hydro­gel matrix, usually reduces the total conductivity due to the dilution effect. Therefore, maintaining conductive channels or networks within the hydro­gel structure is essential. To enhance the conductivity, we propose using benzenesulfonate-terminated F127 (sF127), in which the OH-terminal group of F127 is modified to a benzenesulfonate-terminal bearing a negative charge in the conductive hydro­gels. Consequently, doping pristine F127 micelles with sF127 can create electrostatic interactions with the positively charged PEDOT, facilitating the formation of conducting networks in the micellar crystal hydro­gel. Fig 1 ▸ illustrates the chemical structure and synthesis of sF127. sF127 is synthesized through a two-step process involving tosyl­ation followed by Williamson ether synthesis.

**Figure 2:**
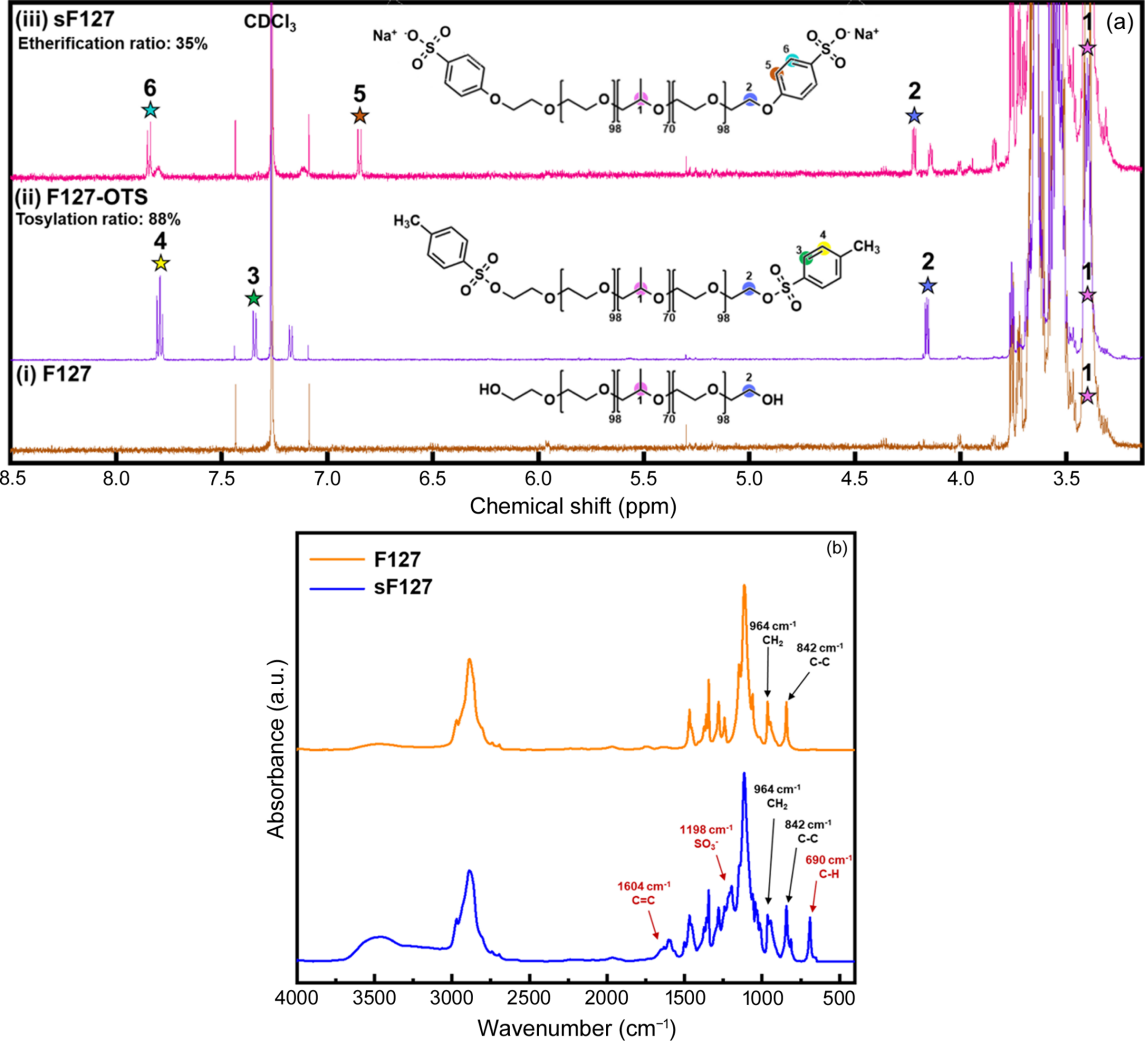
Characterization of sF127 and synthetic intermediates: (*a*) 1H-NMR and (*b*) FTIR spectra.

Fig. 2 ▸ shows the characterization of sF127 and synthetic intermediates via 1H-NMR and FTIR spectra. Fig. 2 ▸(*a*, ii) shows the peaks for the –CH_2_CH_2_O– group of PEO at 3.70–3.74 and 4.14–4.19 p.p.m. (peak 2). Additionally, we observed chemical shifts (δ) at 7.45 p.p.m. (peak 3) and 7.77–7.82 p.p.m. (peak 4), corresponding to protons of the benzene ring of the tosyl­ate (OTs–) group, indicating successful replacement of the terminal hydroxyl group of PEO with electron-withdrawing OTs. The tosyl­ation ratio of F127-OTs was found to be approximately 88%, calculated using the integral area of the tertiary carbon on the PPO chains (peak 1) and the aromatic protons of the OTs group (peak 3 or peak 4).

Fig. 2 ▸(*a*, iii) shows chemical shifts at 6.83–6.86 p.p.m. (peak 5, Ar-H) and 7.78–7.87 p.p.m. (peak 6, Ar-H) that correspond to the position 5 and 6 hydrogens on the benzene ring of the p-HBSA group, respectively. Substitution with p-HBSA led to a shift in the PEO terminal OTs– group signals from 4.14–4.19 p.p.m. to 4.20–4.23 p.p.m. (peak 2). The etherification ratio of the p-HBSA group in sF127 was calculated to be 35%, based on the integral area of the hydrogen signal of the tertiary carbon on PPO (peak 1) and the benzene ring signals of p-HBSA (peak 5 or peak 6). Furthermore, FTIR analysis confirmed the chemical structure. As shown in Fig. 2 ▸(*b*), a comparison of spectral changes in functional groups between F127 and sF127 reveals three absorption peaks at 1604, 690 and 1198 cm^−1^, corresponding to the C=C stretching vibration on the benzene ring of p-HBSA, the C—H out-of-plane bending signal and the antisymmetric vibration of the sulfonic acid group (–SO_3_–) on p-HBSA. These findings confirm the successful synthesis of sF127.

### 3.2. Thermal properties of F_*x*_S_*y*_P hydro­gels

In our strategy, a small amount of sF127 was blended with pristine F127 to introduce a negative charge to the micelle surface, with the expectation of enabling the formation of a conductive network with the positively charged PEDOT in the hydro­gel. The conductive hydro­gels with different blending ratios of F127/sF127 are denoted F_*x*_S_*y*_ and F_*x*_S_*y*_P, where F, S and P represent F127, sF127 and PEDOT:PSS, respectively, and the subscripts *x* and *y* indicate their weight percentages (wt%) in the hydro­gel. Since the critical gel concentration of F127 is 16 wt%, the total content of gel components (*x* + *y*) was maintained at 20 wt% to ensure gel formation. The PEDOT:PSS content was fixed at 0.37 wt%, which was the minimum tested amount that effectively generated an electrical stimulation response.

**Figure 3:**
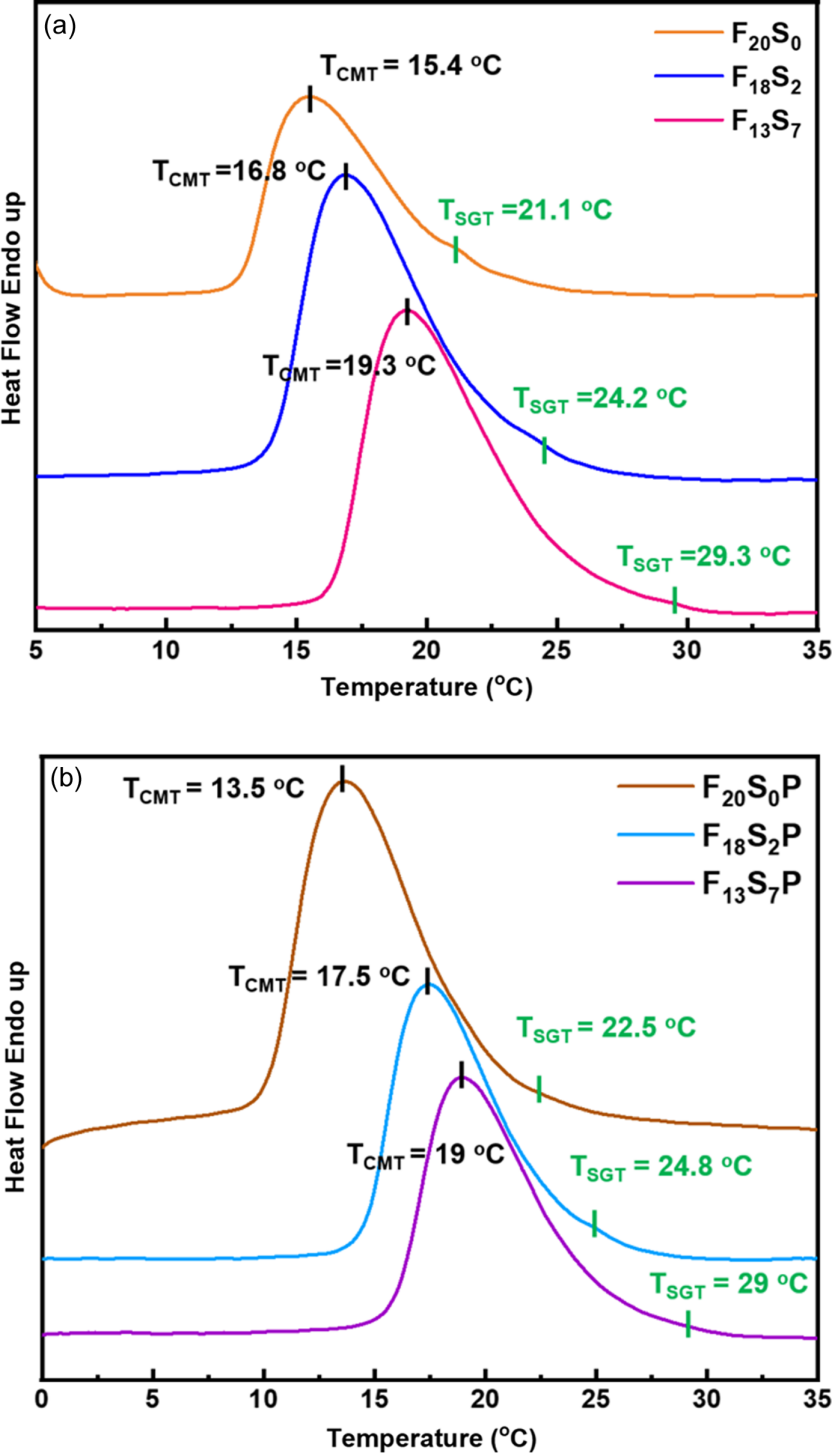
DSC profiles of (*a*) F_*x*_S_*y*_ and (*b*) F_*x*_S_*y*_P hydro­gels during heating.

To study the effect of adding sF127 on the gel formation involving the rearrangement of F127 micelles, Figs. 3 ▸(*a*) and 3 ▸(*b*) show the DSC curves of F_*x*_S_*y*_ and F_*x*_S_*y*_P hydro­gels, respectively, during the heating process. Significant endothermic peaks can be attributed to the critical micellization temperature (*T*_CMT_) due to micellization induced by dehydration of the PPO block in the lower critical solution temperature phase behavior of F127 (Pham Trong *et al.*, 2008 ▸). Additionally, a small shoulder observed on the high-temperature side of the endothermic or exothermic peak indicates the sol-to-gel transition temperature (*T*_SGT_), consistent with rheological measurements in the literature (Pragatheeswaran & Chen, 2013 ▸). In Fig. S2, similar thermal behavior of DSC was also observed during cooling, which implies a reversible sol–gel transition and micellization process. This also indicates that doping with sF127 does not significantly alter the phase behavior of F127. However, as the sF127 content increases, both *T*_CMT_ and *T*_SGT_ gradually rise. This phenomenon can be attributed to the enhanced packing frustration of F_*x*_S_*y*_ micelles with higher sF127 content, requiring higher temperatures for micellization and gelation. This result also indirectly demonstrates that the F127/sF127 blend can form benzenesulfonated micelles.

In comparing F_20_S_0_P and F_20_S_0_, blending pristine F127 with PEDOT:PSS led to a notable reduction in *T*_CMT_ from 15.4 to 13.5°C. The decrease in *T*_CMT_ might account for the stronger hydrogen bonding between water molecules and PSS chains compared with that of the PEO of F127. Thus, slightly different solubility in water results in localized microphase separation to assist the micellization. However, in the cases of F_18_S_2_P and F_13_S_7_P, there is no obvious change in *T*_CMT_ compared with that of F_18_S_2_ and F_13_S_7_. This may be explained by the ability of partially benzenesulfonated micelles of F_*x*_S_*y*_ to increase compatibility with PEDOT:PSS in the hydro­gels. The *T*_SGT_ peaks were also found to be significantly weaker in the F_*x*_S_*y*_P cases, which may be attributed to the lack of ordered packing of F_*x*_S_*y*_ micelles after the addition of PEDOT:PSS. This phenomenon was later confirmed by rheo-SAXS analysis.

For conductivity purposes, the hydro­gel should exhibit good electrical conductivity in order to respond to electric stimulation for drug release. Since the F_13_S_7_ hydro­gel possesses a higher content of benzenesulfonate moieties on the surface of the micelles, a substantial amount of hydro­phobic PEDOT could bind to the benzenesulfonated micelles, leading to inhomogeneity of the hydro­gel. Therefore, we only used 2  wt% sF127 for doping in the electrically responsive hydro­gels (F_18_S_2_P). Conductivity measurements of the hydro­gels using a Swagelok cell indicated that the negatively charged benzene­sulfonate group enables the F_18_S_2_P hydro­gel to achieve a conductivity of 1.44 mS cm^−1^, comparable to that of the PEDOT:PSS aqueous solution (1.66 mS cm^−1^) and three times higher than that of the F_20_S_0_P hydro­gel (0.54 mS cm^−1^). The increase in conductivity of the F_18_S_2_P hydro­gel indicates that PEDOT binding to the benzenesulfonated micelles creates a more effective conductive network within the micellar crystal hydro­gel, providing greater electrical sensitivity compared with the F_20_S_0_P hydro­gel, which lacks the benzene­sulfonate terminal.

### 3.3. Electrically stimulated drug release behavior

To evaluate whether the F_*x*_S_*y*_P hydro­gel can serve as an electrically responsive drug release hydro­gel, we used PX as a model hydro­phobic drug. We encapsulated the PX drug in the F_20_S_0_ and F_18_S_2_ micelles using the thin-film hydration method. Fig. S3 shows the SAXS profiles of the micelles with and without the drug encapsulation. From the fitting model of the core–shell form factor (Guinier & Fournet, 1955 ▸) and sphere structure factor (Percus & Yevick, 1958 ▸) obtained with the *SasView* software (https://www.sasview.org/), we found that the core size of the micelles increased after drug encapsulation (Table S1 of the supporting information). The shell thickness of F_18_S_2_ micelles (6.4 nm) is greater than that of F_20_S_0_ (5.9 nm), which can be attributed to the benzenesulfonated modification in the micelles. When comparing before and after drug encapsulation, the increment in core radius of F_18_S_2_ micelles is greater than that of F_20_S_0_ micelles. We also used the UV–vis absorption intensities of the drug at 354 nm (Fig. S4) and an established calibration curve to quantify the drug encapsulation efficiency and drug loading content as detailed in the supporting information. The encapsulation efficiency and drug loading content of F_18_S_2_/PX micelles were found to be 14.3 and 2.9%, respectively, which are higher than those of the F_20_S_0_/PX micelles (2.3 and 0.5%, respectively). This indicates that the end-group modification loosens the chain packing of the micelles, allowing for the accommodation of more drug molecules.

**Figure 4:**
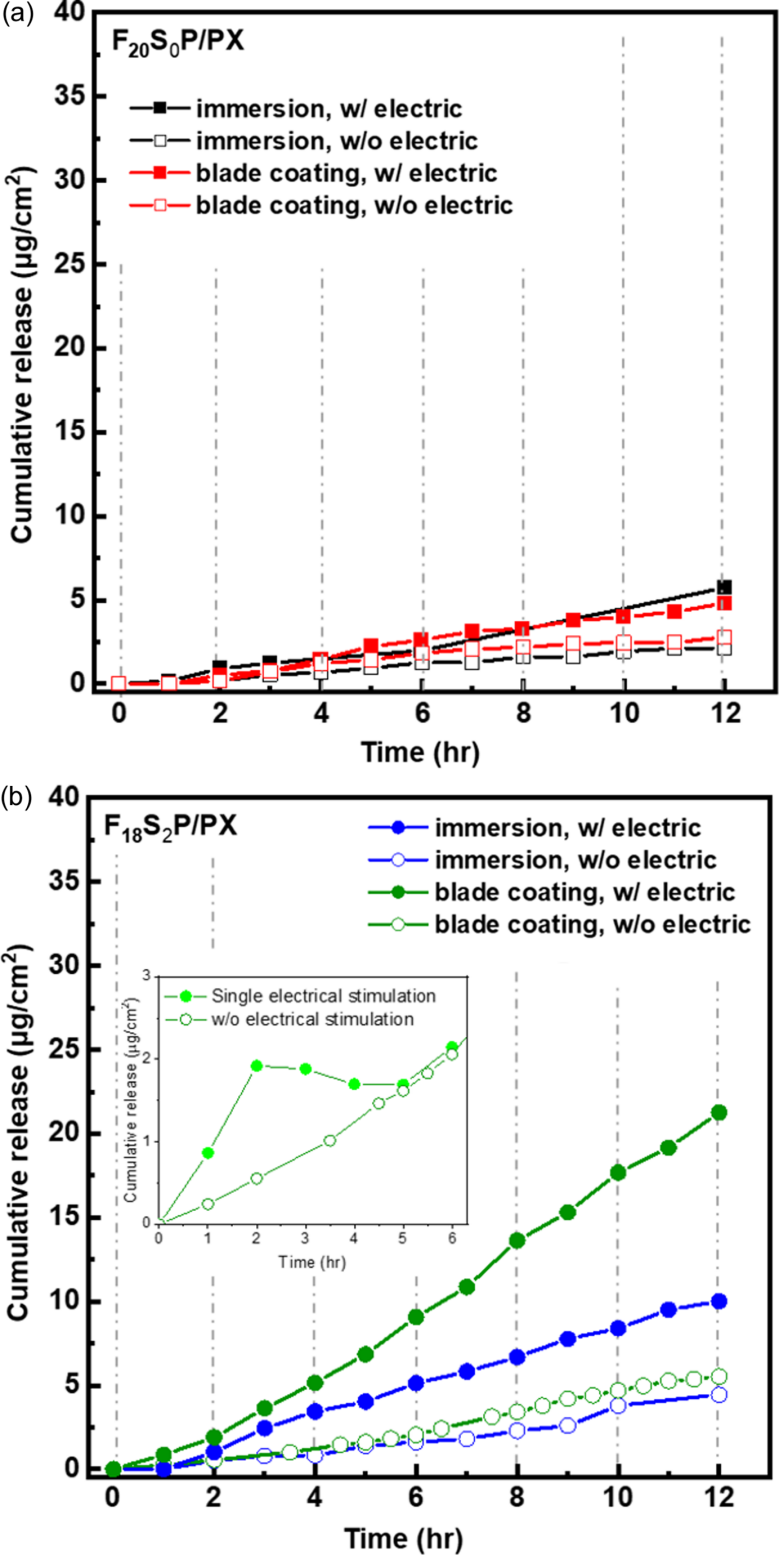
Electrically stimulated drug release curves for (*a*) F_20_S_0_P/PX and (*b*) F_18_S_2_P/PX hydro­gels. The electrical stimulation frequency involves applying a −0.3 V voltage for 60 s every 2 h (marked as dashed lines). The inset in (*b*) represents the drug release curve of the blade-coatingF_18_S_2_P/PX hydro­gel under a single −0.3 V voltage stimulus compared with that without electrical stimulation.

To determine the minimum driving voltage for PX-responsive release from the F_18_S_2_P hydro­gel under *in vitro* electrical stimulation, the prepared samples were subjected to a series of voltages from −0.1 to −1.5 V for 60 s each, as shown in Fig. S5. A significant increase in drug release was observed starting at −0.3 V; therefore, we set −0.3 V as the minimum driving voltage for subsequent experiments. Fig. 4 ▸ shows the effects of two preparation methods (immersion and blade coating) on the drug release of F_20_S_0_P/PX and F_18_S_2_P/PX hydro­gels with and without electrical stimulation. In the immersion method, carbon felt electrodes are prepared by submerging them in the encapsulated drug hydro­gels (F_20_S_0_P/PX or F_18_S_2_P/PX). In contrast, in the blade-coating method, the encapsulated drug hydro­gels are applied onto the carbon felt through repeated spreading with a blade. Drug release curves can be obtained using a single electric stimulus lasting for 60 s and then monitoring as it returns to the baseline (without electrical stimulation) for 2 h, as shown in the inset of Fig. 4 ▸(*b*). Therefore, we conducted the electrically stimulated drug release test by applying a −0.3 V electric stimulus for 60 s in cycles every 2 h to measure drug release curves (Fig. 4 ▸).

In Fig. 4 ▸(*a*), the drug release by the F_20_S_0_P/PX hydro­gel shows no significant difference between the two preparation methods, although electrical stimulation does slightly increase the amount of drug released. This phenomenon confirms that micelles lacking benzene­sulfonate modification do not display significant electrically stimulated drug release behavior. In contrast, when the voltage is applied, the drug release from the F_18_S_2_P/PX hydro­gel dramatically accelerates, and it continues to increase over time [Fig. 4 ▸(*b*)]. Furthermore, the F_18_S_2_P/PX hydro­gel exhibits significant drug release for both preparation methods, with a particularly strong response to applied voltage. This suggests that the voltage-triggered redox process may generate interactive forces between the F_18_S_2_ micelles and PEDOT, potentially causing micelle deformation and the subsequent release of the loaded drug PX. Notably, the drug release amount from the F_18_S_2_P/PX hydro­gel prepared using the blade-coating method is twice that of the immersion method, indicating that the shear force during the blade-coating process has a significant impact on the conducting network structure within the micelles of the F_18_S_2_P hydro­gel.

### 3.4. Viscoelastic micellar crystal hydro­gels under LAOS

**Figure 5:**
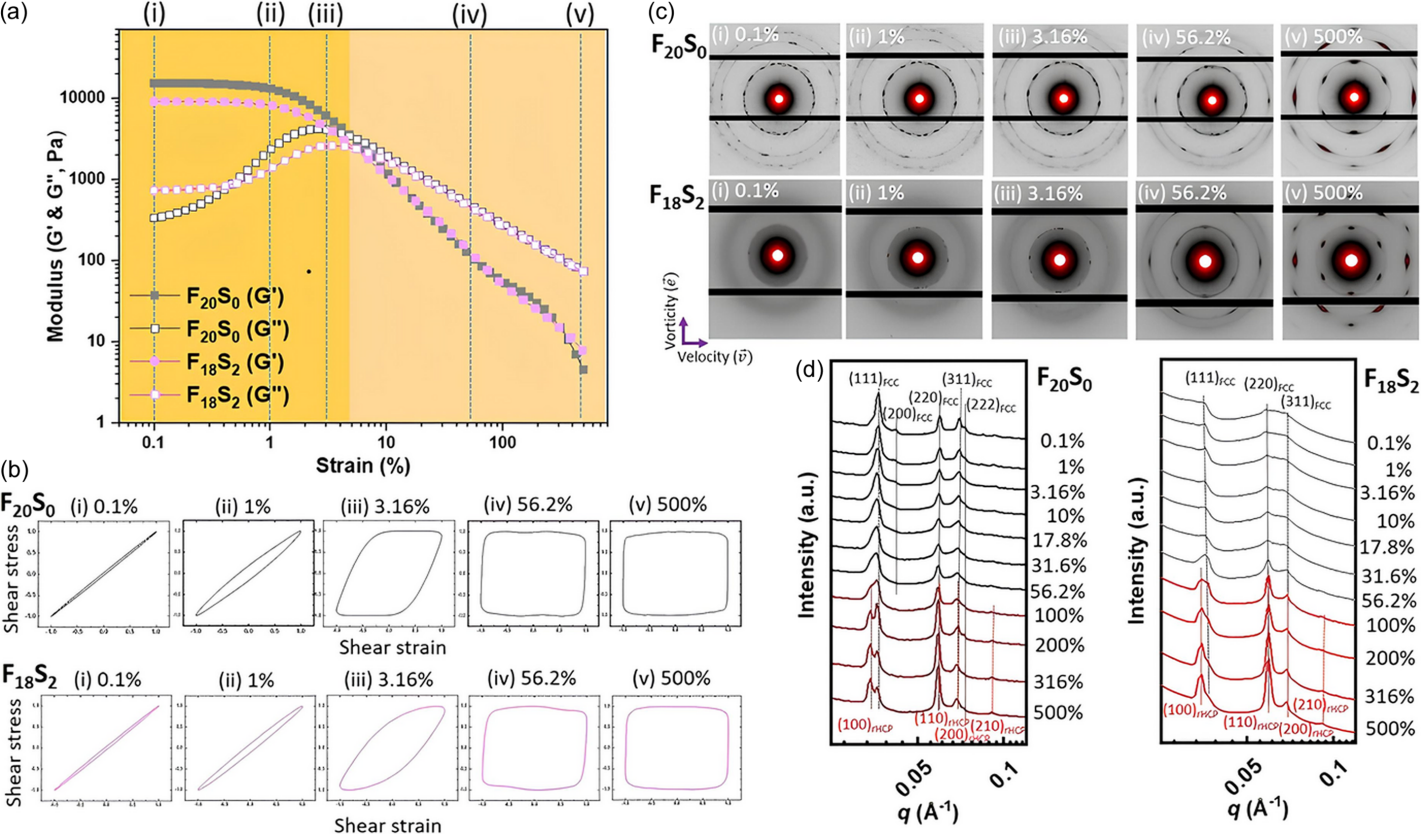
Rheo-SAXS data of F_20_S_0_ and F_18_S_2_ hydro­gels under LAOS: (*a*) *G*′ and *G*′′ curves, (*b*) elastic Lissajous–Bowditch curves, (*c*) representative 2D SAXS patterns, and (*d*) 1D SAXS profiles (full azimuthal integration).

By investigating electrically stimulated drug release, we found that preparation of conductive F_18_S_2_P hydro­gels using the blade-coating process significantly enhances drug release. To understand how the blade-coating process influences the structure of the conductive hydro­gel and subsequently affects drug release, we utilized the rheo-SAXS experiment combined with LAOS methodology. The aim was to investigate the rheological behavior and order–order phase transitions of the micellar crystal hydro­gels (including F_20_S_0_, F_18_S_2_, F_20_S_0_P and F_18_S_2_P) under LAOS, thereby elucidating the impact of the blade-coating process stress on drug release performance in hydro­gels. Therefore, before investigating conducting hydro­gels, we first needed to understand whether the rheological properties and the order–order phase transitions of the micelles had undergone significant changes with and without the benzenesulfonated modification for the micelles, as shown in Fig. 5 ▸.

Fig. 5 ▸(*a*) presents the storage modulus (*G*′) and loss modulus (*G*′′) as functions of strain amplitude sweep for both the F_20_S_0_ and the F_18_S_2_ hydro­gels. A progressive drop in the storage modulus can be seen, which shows that shear thinning happens as the strain amplitude rises for both cases. As the strain is lower than approximately 5%, *G*′ > *G*′′ indicates a gel state. Conversely, at higher strains (>5%), *G*′ < *G*′′ signifies that a gel-to-sol transition has occurred. Both the F_20_S_0_ and the F_18_S_2_ hydro­gels exhibit the same gel-to-sol transition at approximately 5% strain. However, the modulus of F_18_S_2_ is smaller than that of F_20_S_0_ only in the gel state, while both exhibit a nearly identical modulus in the sol state. These findings suggest that the benzenesulfonated modified micelles of F_18_S_2_ hydro­gel possess lower elasticity. Furthermore, the gel-to-sol transition is reflected in the hysteresis loops of energy dissipation observed in the elastic Lissajous–Bowditch curves [Fig. 5 ▸(*b*)], which visually illustrate the material’s response during an amplitude sweep (Hyun *et al.*, 2011 ▸). At low strain amplitudes (<5%), a straight line indicates elastic behavior, whereas an ellipse suggests behavior approaching viscoelasticity. Due to the hydro­gel’s inability to endure extreme deformation and release energy, the area of the hysteresis circle likewise becomes rectangular at higher strain amplitudes (>5%), signifying ideal plastic behavior.

Fig. 5 ▸(*c*) shows the representative 2D SAXS patterns corresponding to various strain amplitudes. As the strain amplitudes increase, the micellar crystals of both the F_20_S_0_ and the F_18_S_2_ hydro­gels clearly transition from a polycrystalline to a single-crystalline-like structure. 1D SAXS profiles at different strain amplitudes are summarized in Fig. 5 ▸(*d*) for the F_20_S_0_ and F_18_S_2_ hydro­gels. At lower strains (<40%), the micellar crystal of the F_20_S_0_ hydro­gel exhibits a face-centered cubic (FCC) structure with the lattice parameter *a*_FCC_ = 17.16 nm, demonstrated by a series of diffraction peaks with a positional ratio of 1:(4/3)^1/2^:(8/3)^1/2^:(11/3)^1/2^:(12/3)^1/2^, corresponding to the 111, 200, 220, 311 and 222 diffraction peaks, respectively. As the strain amplitude exceeds 40%, three new diffraction peaks with a positional ratio of 1:3^1/2^:2 emerge, overlapping with the FCC diffraction peaks and gradually dominating the diffraction pattern. This can be attributed to the original *ABC* layer-stacked FCC structure undergoing interlayer sliding under higher strain amplitudes, leading to the induction of a randomly hexagonal close-packed (rHCP) structure with the lattice parameter *a*_rHCP_ = 15.8 nm. The rHCP is characterized by random layer stacking, with each layer consisting of 2D, hexagonally packed micelles. Therefore, when X-ray beams are perpendicular to the rHCP layer plane, sixfold diffraction spots can be observed in the radial SAXS pattern [Fig. 5 ▸(*c*)] at the 200 and 500% strains. In Fig. S6, the tangential SAXS patterns under the 500% strain also demonstrate the rHCP phase for F_20_S_0_ and F_18_S_2_. Similar rHCP structures in F127 micellar crystals were observed in rheological small-angle neutron scattering measurements (Jiang *et al.*, 2007 ▸; López-Barrón *et al.*, 2012 ▸).

However, the F_18_S_2_ hydro­gel exhibits broad and weak diffraction peaks at low strain amplitudes, while sharp diffraction peaks with a positional ratio of 1:3^1/2^:2 emerge only as the strain amplitudes increase. This suggests that the partially benzenesulfonated groups on the micelle surface could induce packing frustration and hinder the self-assembly growth of micellar crystals. Therefore, at low strain amplitudes, short-range-ordered FCC-like packed micelles are observed, while higher strain amplitudes are required to promote the formation of the rHCP structure in the F_18_S_2_ hydro­gel.

**Figure 6:**
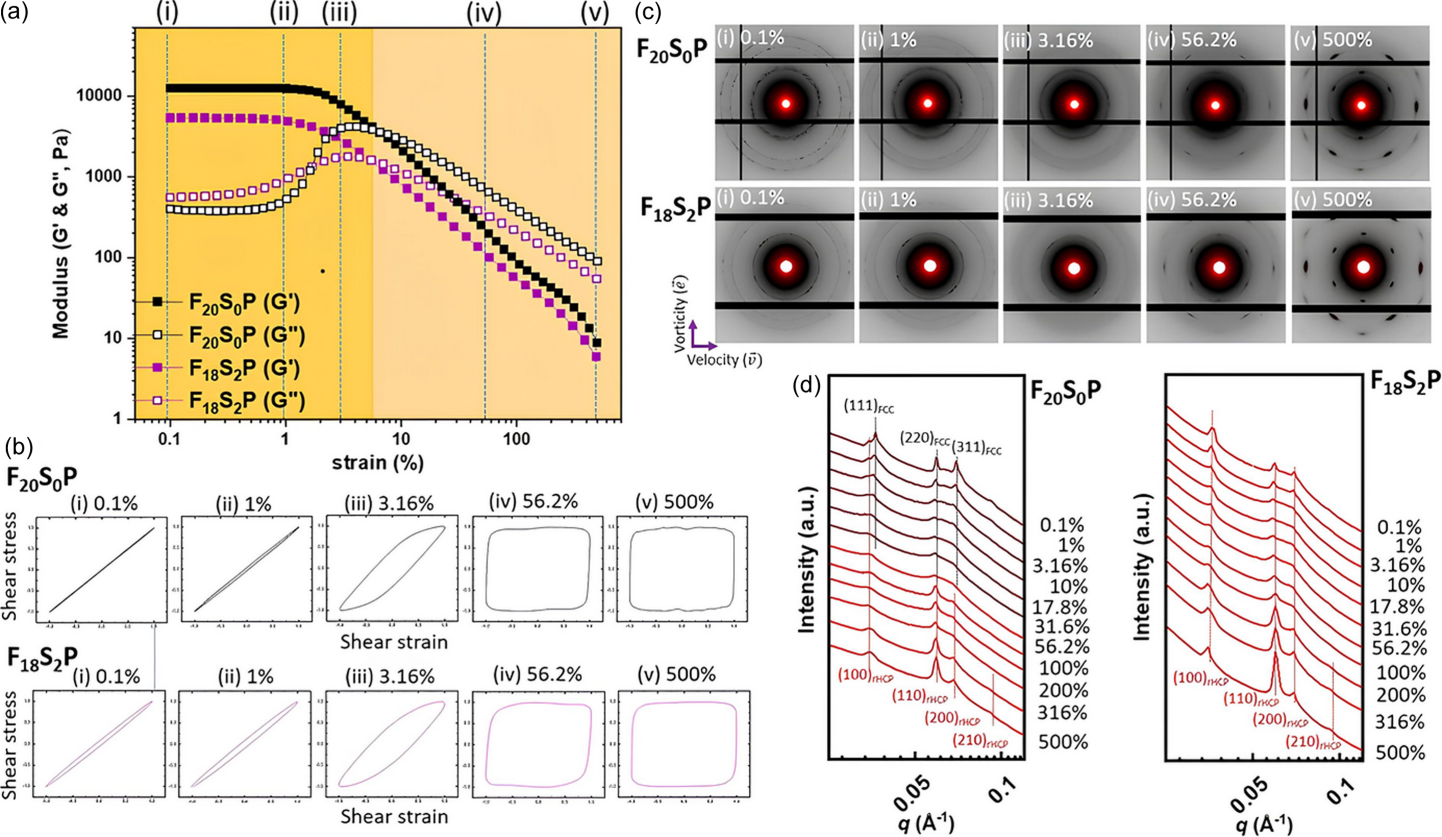
Rheo-SAXS data of F_20_S_0_P and F_18_S_2_P hydro­gels under LAOS: (*a*) *G*′ and *G*′′ curves, (*b*) elastic Lissajous–Bowditch curves, (*c*) representative 2D SAXS patterns, and (*d*) 1D SAXS profiles (full azimuthal integration).

Fig. 6 ▸ presents the *G*′ and *G*′′ modulus curves, Lissajous–Bowditch curves, and SAXS patterns under LAOS for both of the conducting hydro­gels of F_20_S_0_P and F_18_S_2_P. In the F_20_S_0_P hydro­gel, we observed that the shapes of the Lissajous–Bowditch curves and the gel-to-sol transition behavior are similar to those of the F_20_S_0_ hydro­gel, indicating that the addition of PEDOT:PSS does not affect the viscoelastic properties. However, from the diffraction intensity and peak width in the SAXS profiles of F_20_S_0_P [Fig. 6 ▸(*d*)], it is evident that the crystallinity and grain size significantly decrease compared with the F_20_S_0_ hydro­gel. A closer examination of the radial and tangential SAXS patterns [Fig. 6 ▸(*c*) and S6] also reveals that, in the low-strain range, FCC and rHCP phases coexist, but as the strain increases, the FCC diffraction peaks gradually disappear and transform into a single phase of the rHCP phase, starting at 56.2%. This result suggests poor compatibility between unmodified micelles and PEDOT:PSS. This is also the main reason why the F_20_S_0_P/PX hydro­gel cannot effectively achieve electrically stimulated drug release [Fig. 4 ▸(*a*)].

However, the F_18_S_2_P hydro­gel shows consistent positioning of the first diffraction peak and the peak position ratio is 1:3^1/2^:2 across varied strain amplitudes, suggesting the absence of any phase transition during LAOS [Fig. 6 ▸(*d*)]. This finding suggests that the negatively charged PEDOT effectively binds to the positively charged benzenesulfonated groups on the micelle surface through electrostatic interactions, resulting in randomly close-packed micelles. This is also the reason for the significantly reduced modulus of F_18_S_2_P [Fig. 6 ▸(*a*)]. These observations are consistent with our previous experimental and theoretical simulation studies, which demonstrated that photonic colloidal aggregates with maximally random jammed packing can spontaneously form through electrostatic attractions between PEDOT:PSS and colloids (Chuang *et al.*, 2024 ▸). Additionally, as strain exceeds 6%, the gel-to-sol transition initiates, allowing for sliding and alignment, and ultimately forming the highly ordered rHCP phase, as shown by the radical and tangential SAXS patterns observed under high strain [Fig. 6 ▸(*c*) and S6]. It can be inferred that the F_18_S_2_P hydro­gel, under the action of shear force, enables a more uniform distribution of PEDOT around the micelles, forming a conductive network that facilitates electrically stimulated drug release.

**Figure 7:**
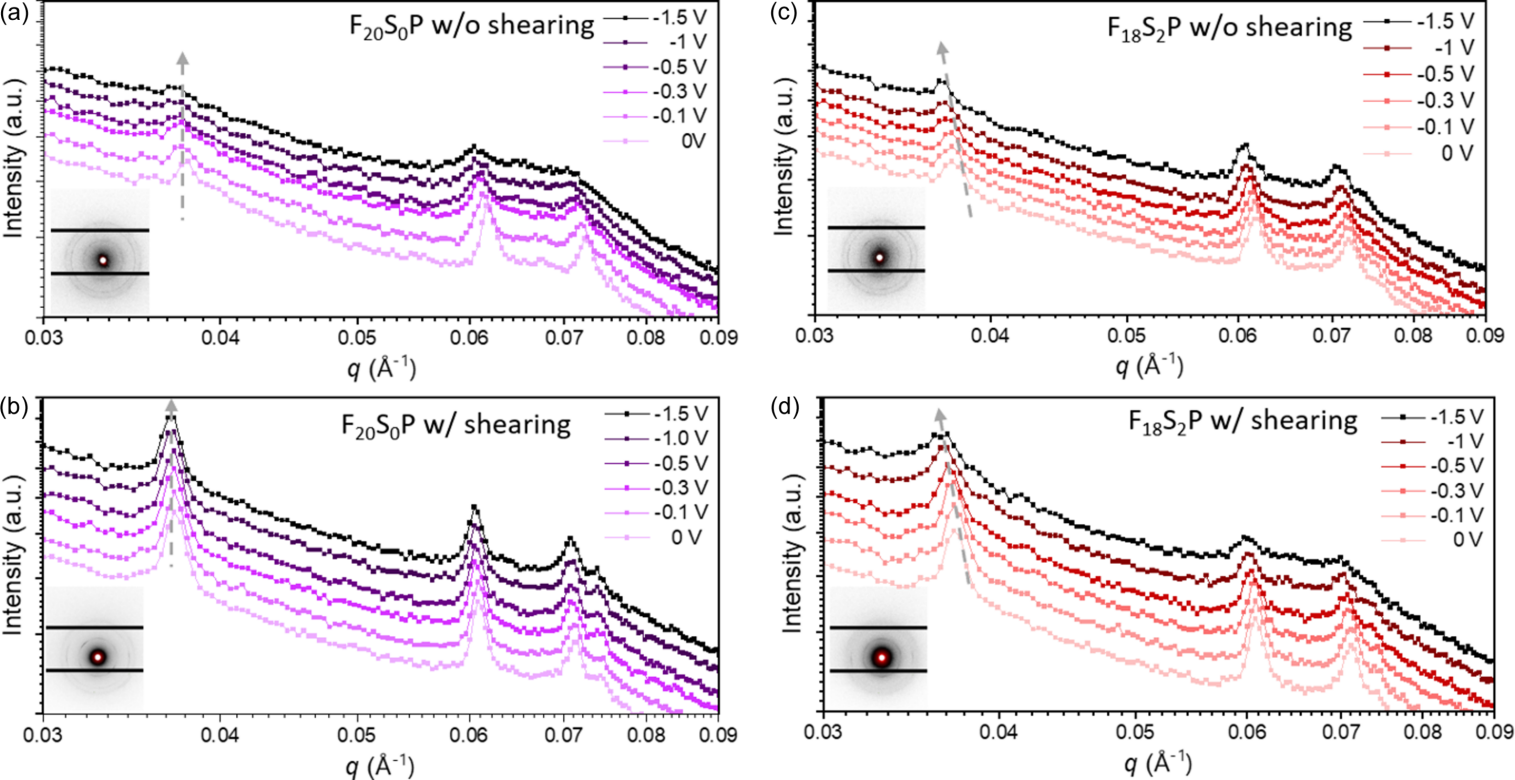
*In situ* E-SAXS profiles of the hydro­gels under a series of voltages: F_20_S_0_P hydro­gel (*a*) without and (*b*) with shearing; F_18_S_2_P hydro­gel (*c*) without and (*d*) with shearing. The insets show the 2D SAXS patterns of the various hydro­gels.

In addition to investigating the micelle packing behavior of the hydro­gels under shear using rheo-SAXS, we further employed *in situ* E-SAXS to clarify the electrical stimulation of the F_*x*_S_*y*_P hydro­gels. The E-SAXS setup consisted of two copper electrodes [Fig. S1(*b*)], between which the hydro­gel samples were either directly placed or applied using a blade-coating method. Various voltages were then applied across the electrodes while SAXS measurements were conducted simultaneously. Fig. 7 ▸ shows the E-SAXS profiles of F_20_S_0_P and F_18_S_2_P hydro­gels under different applied voltages, with and without prior shear treatment. As illustrated in Figs. 7 ▸(*a*) and 7 ▸(*b*), the diffraction peaks of the F_20_S_0_P hydro­gels exhibit negligible changes on application of electric fields, regardless of the shear treatment. This result is consistent with the pristine F127 (F_20_) hydro­gels of non-conductive nature, where no noticeable shift in diffraction peaks occurs under applied voltage (Fig S7).

In contrast, as shown in Figs. 7 ▸(*c*) and 7 ▸(*d*), the diffraction peaks of the F_18_S_2_P hydro­gels gradually shift toward lower *q* values with increasing applied voltage, indicating that the micelle packing becomes more expanded, leading to a larger crystalline lattice spacing under the influence of the electric field. This pronounced structural change under the electric fields can be attributed to the superior electrical conductivity of the F_18_S_2_P hydro­gel, which reaches approximately 1.44 mS cm^−1^, compared with the F_20_S_0_P hydro­gel with a conductivity of only 0.54 mS cm^−1^. These findings directly demonstrate the electric-field responsiveness of the sulfonated micelles of the F_18_S_2_P hydro­gels, with the effect being more pronounced in the shear-treated hydro­gels. This also provides a rational explanation for the enhanced electrically stimulated drug release observed in the F_18_S_2_P system (as shown in Fig. 4 ▸), where blade-coated samples exhibit significantly higher drug release efficiency than those prepared via immersion.

The structural and rheological characterizations presented in this study offer direct insight into the design principles underlying the development of electrically responsive F_*x*_S_*y*_P hydro­gels. Through rheo-SAXS analysis, we demonstrated that micellar crystal hydro­gels undergo significant FCC-to-rHCP transitions under shearing. This phase transition was especially pronounced in the F_18_S_2_ and F_18_S_2_P systems, where sulfonated end groups not only increased electrostatic compatibility with PEDOT:PSS but also induced packing frustration, lowering micellar elasticity and enabling better alignment under mechanical stress. Interestingly, the impact of the processing method – specifically blade-coating versus immersion – on drug release efficiency was also evident. The blade-coated hydro­gels showed notably enhanced electrically stimulated drug release, with the F_18_S_2_P/PX system achieving nearly double the release compared with immersion-prepared samples (Fig. 4 ▸). This enhancement is attributed to the shear stress introduced during the blade-coating process, which mirrors the mechanical conditions applied in rheo-SAXS.

**Figure 8:**
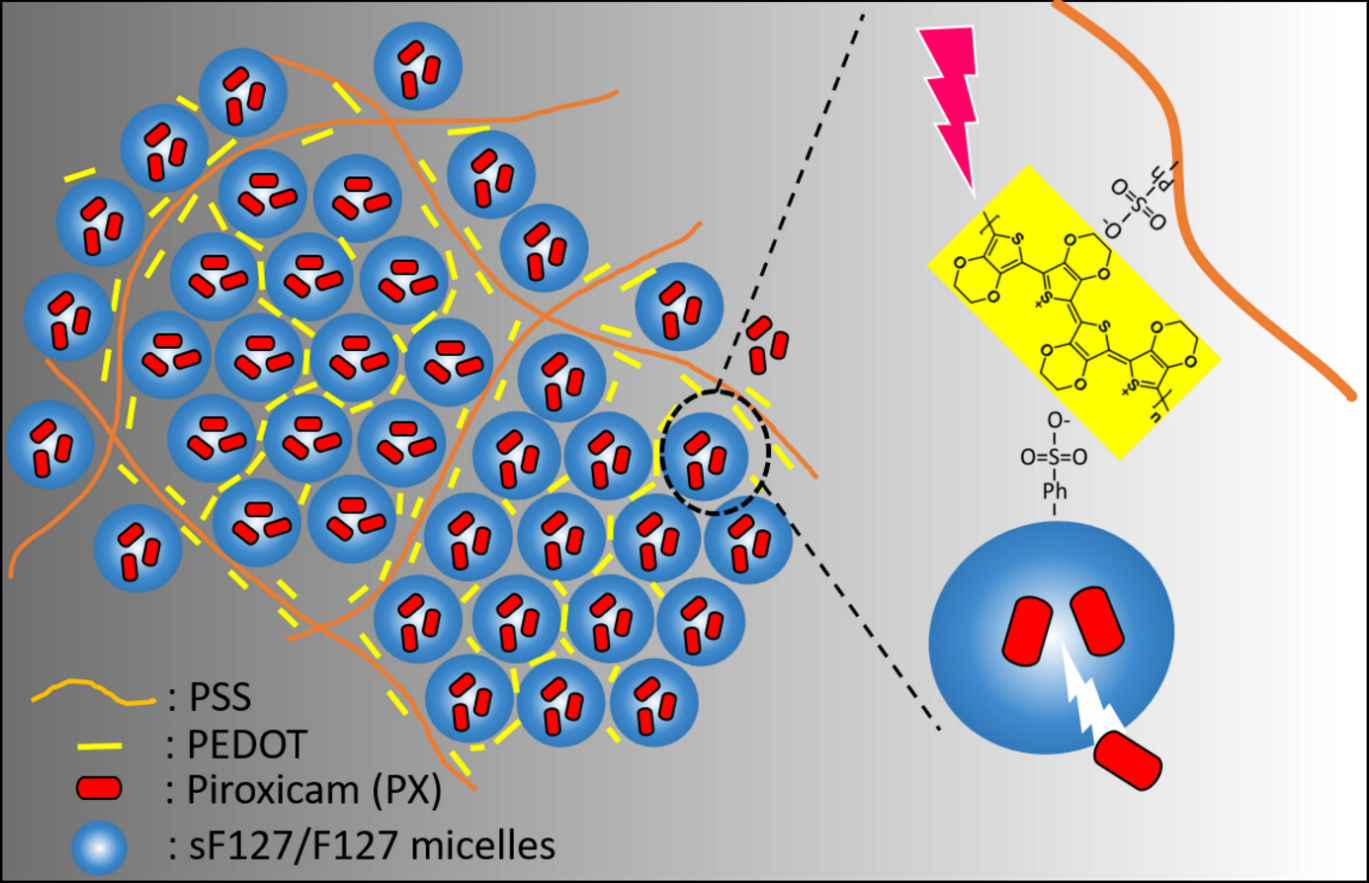
Schematic of the nanostructural features of the F_18_S_2_P hydro­gel for the electrically stimulated drug release.

By integrating the rheo-SAXS and E-SAXS findings with blade-coating processing insights, we identify a clear structure–function relationship that governs the performance of these hydro­gels as illustrated in Fig. 8 ▸. The shear-induced structural transition creates conductive nanonetworks by aligning PEDOT uniformly around sulfonated micelles. This conductive architecture is critical in transmitting voltage-induced redox responses efficiently throughout the hydro­gel matrix. Upon electrical stimulation, PEDOT’s redox switching modulates its charge distribution and interfacial interactions, leading to local deformation of micellar aggregates and facilitating the drug expulsion (Puiggalí-Jou *et al.*, 2020 ▸; Molina *et al.*, 2018 ▸; Kleber *et al.*, 2019 ▸). These morphological and electrical changes, synchronized by controlled micelle alignment, are crucial for the observed enhancement in on-demand drug release.

## 4. Conclusions

This study successfully developed an innovative electrically responsive hydro­gel based on PEDOT:PSS and partially benzenesulfonated Pluronic F127 micelles (*i.e.* F_18_S_2_P). rheo-SAXS and E-SAXS analyses confirmed that the introduction of negatively charged benzene­sulfonate groups enhanced the formation of conductive networks within micellar crystals, significantly improving the hydro­gel’s drug encapsulation efficiency and electrical responsiveness, particularly when using blade-coating methods. These results establish a structure–processing–function relationship, demonstrating how shear-mediated micellar alignment enables more effective electroresponsive behavior. This work offers a mechanistic foundation for designing high-performance stimulus-responsive hydro­gels for on-demand therapeutic applications. 

## Supplementary Material

Supporting figures and table. DOI: 10.1107/S1600576725002808/uu5011sup1.pdf

## Data Availability

Details on the configuration of the Couette cylinder cell used for the rheo-SAXS and DSC analyses and the fitting parameters for SAXS are included in the supporting information.
