# Supplementary material for: Rheo-SAXS study on electrically responsive hydro­gels with shear-induced conductive micellar networks for on-demand drug release
Source: J Appl Crystallogr. 2025 Apr 25;58(Pt 3):909–18. doi: 10.1107/S1600576725002808 (PMC12135972; doi:10.1107/S1600576725002808)
Supplement: Supplementary file 1 [file j-58-00909-sup1.pdf]

JOURNAL OF APPLIED CRYSTALLOGRAPHY

Volume 58 (2025)

Supporting information for article:

Rheo-SAXS study on electrically responsive hydrogels with shear-induced conductive micellar networks for on-demand drug release

Thuy Thien Ngan Vo, Yi-Wei Chang, Chun-Jen Su, U-Ser Jeng, Chih-Chia Cheng, Ya-Sen Sun and Wei-Tsung Chuang

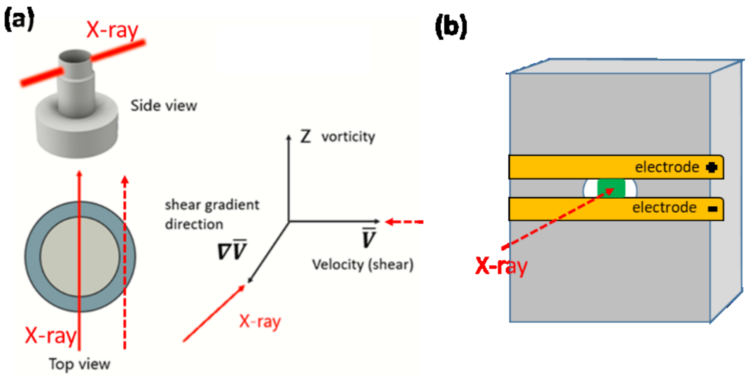

Figure S1. Schematic diagrams of (a) the Couette cylinder cell used for the Rheo-SAXS measurement and (b) applied electric device for the E-SAXS measurement.

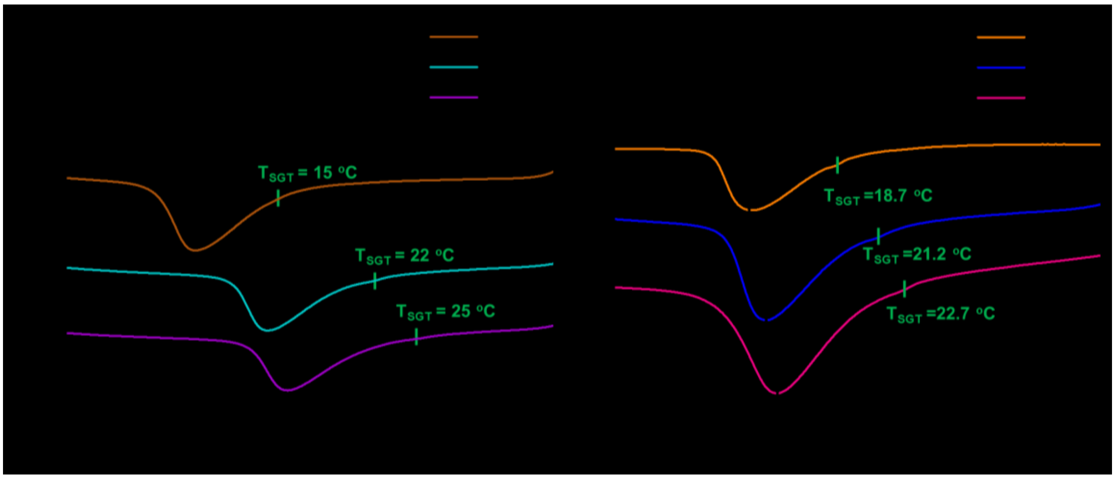

Figure S2. DSC profiles of (a) F_xS_y hydrogels and (b) F_xS_yP hydrogels during a cooling process.

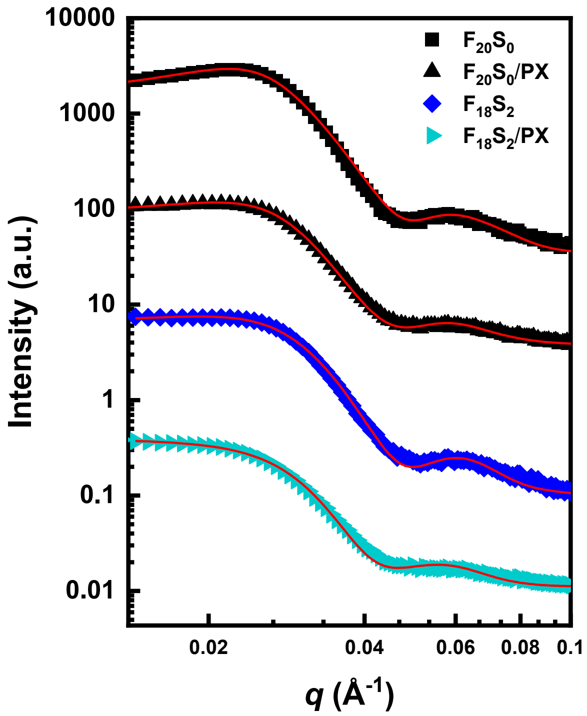

Figure S3. SAXS profiles for dilute solutions of $F_{20}S_0$, $F_{20}S_0$/PX, $F_{18}S_2$ and $F_{18}S_2$/PX. Red lines are fitted by the model of core-shell form factor and hardsphere structure factor.

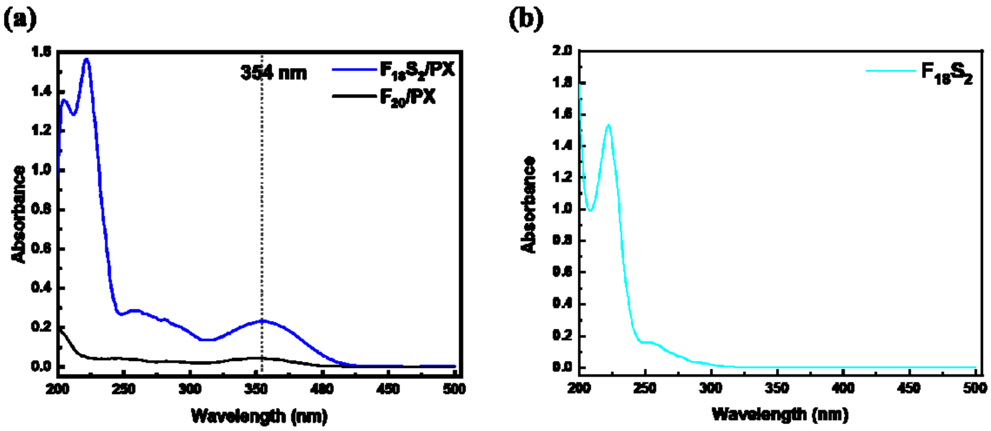

Figure S4. UV-Vis spectra of (a)$F_{20}S_0$/PX and $F_{18}S_2$/PX (b) $F_{18}S_2$.

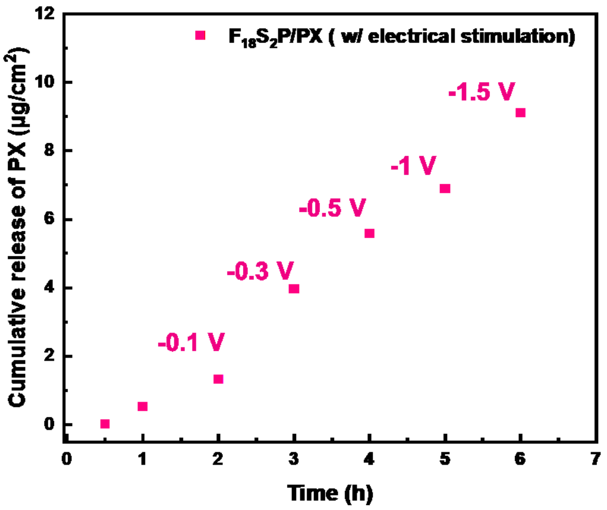

Figure S5. Drug release profiles of the $F_{18}S_2P$/PX hydrogel under electrical stimulations with various applied voltages.

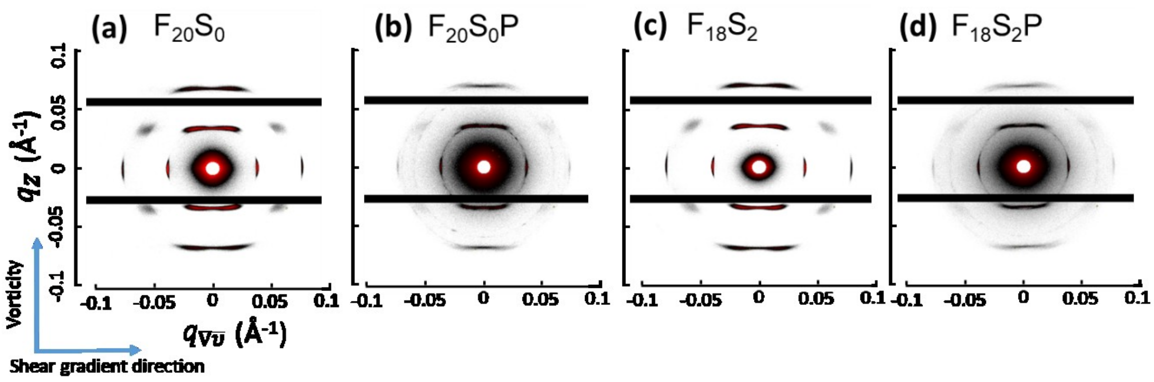

Figure S6. Tangential SAXS patterns under 500% strains for (a) $F_{20}S_0$ (b) $F_{20}S_0P$ (c) $F_{18}S_2$ (d) $F_{18}S_2P$.

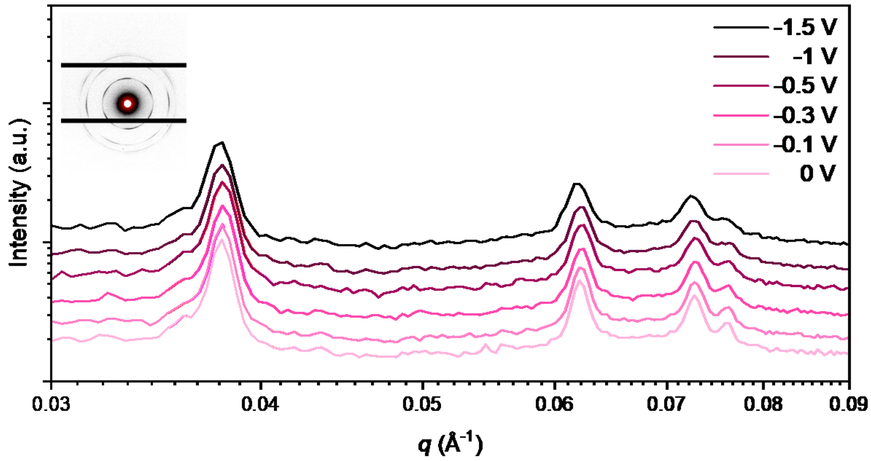

Figure S7. In-situ E-SAXS profiles of the pristine F127 hydrogel under a series of voltages. The inset shows the 2D SAXS patterns.

Table S1. SAXS model fitting parameters

Sample	$F_{20}S_0$	$F_{20}S_0$/PX	$F_{18}S_2$	$F_{18}S_2$/PX
Model	Core-shell form factor + hardsphere structure factor			
Core radius (nm)	2.6	2.8	2.3	2.9
Shell thickness (nm)	5.9	5.9	6.4	6.4
Volume fraction	0.21	0.18	0.16	0.14
